# The Effectiveness of Cognitive Behavioral Therapy versus Notched Sound Therapy in Adults with Chronic Subjective Tinnitus and Normal Hearing

**DOI:** 10.1055/s-0044-1788000

**Published:** 2024-10-25

**Authors:** Soha Abdelraouf Mekki, Mohammed Gamal Sehlo, Usama Mahmoud Youssef, Ola Abdallah Ibraheem, Mai Ragab Ghazaly

**Affiliations:** 1 Department of Otorhinolaryngology, Faculty of Medicine, Zagazig University, Zagazig, Egypt; 2 Department of Psychiatry, Faculty of Medicine, Zagazig University, Zagazig, Egypt

**Keywords:** tinnitus, cognitive behavioral therapy, notched sound therapy, loudness matching

## Abstract

**Introduction** Tinnitus can be distressing, and it affects the quality of life (QoL) through psychological and cognitive impairments. Cognitive behavioral therapy (CBT) and notched sound therapy (NST) are tinnitus management approaches aiming to reduce symptoms and improve QoL via two different mechanisms. The CBT attains the cognitive principle, whereas the NST initiates tinnitus habituation.

**Objective** To evaluate the effect of CBT and NST and compare their results in the management of chronic subjective tinnitus.

**Methods** The present prospective study involved 64 adults with normal hearing and chronic subjective tinnitus. They were subjected to history taking, basic audiological evaluation, and extended high-frequency audiometry at 10 and 12.5 kHz. The participants were divided into two equal groups, the first treated with CBT and the second treated with NST. The psychoacoustic measures of tinnitus and the Arabic Questionnaire for Tinnitus Reaction (Arabic-QTR) were used to monitor the outcomes of both therapies.

**Results** Both groups showed significant reduction in tinnitus severity according to the Arabic-QTR and tinnitus loudness matching. Improvement in the Arabic-QTR was better in the CBT group, while tinnitus loudness improvement was better in the NST group.

**Conclusion** Both CBT and NST are effective in the management of chronic subjective tinnitus. In a comparison of the effect of the two therapies, CBT was found to be more effective in decreasing tinnitus-related distress, whereas SNT was found to be more helpful in reducing tinnitus loudness.

## Introduction

Tinnitus is the perception of sound without an external sound generator, in most cases. It may be heard in one or both ears, the center of the head, or even away from the head. 1 Tinnitus can be classified into two types: objective and subjective. Subjective tinnitus is more common and perceived only by the individual. It is a highly complex condition with a multi-factorial origin, heterogeneous patient proﬁles, and caused by anomalous activity in the auditory system. 2

The importance of tinnitus could be related to the relatively high prevalence rates, which range from 7.1 to 14.6% according to the National Center for Health Statistics 3 and from 10 to 19% according to Gilles et al. 4 Another important issue is the impact of tinnitus on quality of life (QoL), which varies from non-bothersome to bothersome. 2 Bothersome tinnitus causes negative emotional responses such as anxiety and depression, which markedly affect QoL. In addition, it causes disabling psychological and cognitive manifestations such as insomnia and difficulties in concentration, communication, and social interaction. 5

The relationship involving the limbic system, the autonomic system, and the auditory system has been explained by the neurophysiological model proposed by Jastreboff; 6 the model hypothesizes that tinnitus develops through the generation of abnormal neural activities in the auditory pathways that are processed as sourceless sound waves by the peripheral auditory system and move through the auditory pathways to the primary auditory cortex and other cortical areas. The abnormal neural activities are evaluated subconsciously and consciously. If they are evaluated as representing a neutral event, they will not be perceived consciously. However, if the neural activities are evaluated negatively or as unknown, they will be classified as potentially unpleasant and/or dangerous, which activates the limbic and autonomic nervous systems and subsequently generates negative reactions such as annoyance, anxiety, panic, and concentration disturbance. These reactions have two-way connections to the limbic system, autonomic nervous system, and the auditory cortex and other cortical areas.

Therefore, there is a need for prompt tinnitus management. Uniform treatment can be challenging due to the heterogeneous and highly variable nature of tinnitus. Over the past few decades, numerous approaches have been developed to manage tinnitus. The treatments commonly administered include habituation therapy, antidepressants, hearing aids, transcranial magnetic stimulation, and tinnitus retraining therapy. 2 In addition, the cognitive behavioral therapy (CBT) 7 8 9 and notched sound therapy 10 11 12 13 approaches have currently gained much interest.

An integrative and pragmatic therapy, CBT aims at modifying dysfunctional behaviors and beliefs to reduce symptoms, increase daily life functioning, and ultimately promote recovery. 14 The CBT approach includes a combination of several elements, such as education, counseling, exposure, relaxation, and hearing rehabilitations. There is strong evidence favoring tinnitus treatment by CBT, 8 which has been applied in tinnitus research for decades and the results of the eﬀectiveness vary in decreasing tinnitus severity/distress, tinnitus-related fear, tinnitus disability, tinnitus-related cognitive problems, and in improving QoL. 5

On the other hand, sound therapy is used in different ways, such as the neuromonics approach (music combined with broadband noise, providing an equalized stimulation), 15 tinnitus-masking therapy, 16 customized music stimulation, 17 and notched sound therapy (NST). 10 11 18 These approaches aim at improving tinnitus not by treating its causes, but simply by helping to manage its consequences. 2 Listening to NST can introduce a functional deafferentation of auditory neurons corresponding to the eliminated frequency band. This could be explained on the basis that deprivation of auditory input in the tinnitus-matched frequency range results in the long-term depression of auditory neurons matching the tinnitus frequency. 19 20 Consequently, the notched sound no longer stimulates the cortical area corresponding to the tinnitus frequency, and it can effectively reduce tinnitus loudness and tinnitus-related auditory cortex activity, 21 22 providing amelioration on thoughts, emotions, concentration, sleep, and tinnitus annoyance. 23

To the best of our knowledge, there are no previous studies on the effect of CBT versus NST and when to incorporate each of them in adults with chronic subjective tinnitus. Therefore, the current study was designed to evaluate the effect of CBT and NST and to compare their outcomes in adults with chronic subjective tinnitus and normal hearing using the psychoacoustic measures of tinnitus (tinnitus pitch and loudness matching) and the newly developed Arabic Questionnaire for Tinnitus Reaction (Arabic-QTR), a self-assessment scale with high validity and reliability. 24 Based on the patient's main complaint (such as tinnitus loudness or distress) and the outcomes of the assessment measures, a clue could be given regarding when to apply CBT or NST.

## Methods

### Participants

The present prospective cohort study involved adults (aged between 18 and 50 years) of both genders with tinnitus complaints of variable severity. The sample was calculated to contain 64 subjects, using the Open-Epi (open source) application at a confidence level of 95% and power of 80%. The sample was randomly divided (simple random method) into 2 groups of 32 subjects each: the first group received CBT, while the second group received NST of notched white noise. The inclusion criteria were as follows: normal hearing threshold in the frequency range between 250 Hz and 8 kHz (up to 25 dB HL), 25 with excellent word recognition score (WRS: 88–100%), unilateral or bilateral chronic subjective tinnitus (for ≥ 6 months) 2 of tonal character (whistling or beeping sounds), as the notch of the NST is centered around pitch match of tonal tinnitus, 26 pitch perception of tinnitus is subjectively stable (stable tinnitus when the patient has experienced it for 6 months or more without change in pitch, loudness, laterality, and nature), 2 and normal neurological examinations.

Participants were excluded from the study when they presented: objective or pulsatile tinnitus, because they reflect an underlying pathology that needs further investigation and management; external and/or middle-ear pathology; neurological disorders, such as dementia, intellectual disability, or past history of major mental illness (psychosis, bipolar disorder, depression, anxiety disorder, or the use of psychotropic medication); and history of ototoxic medications and substance abuse. The study began after we obtained approval from the Institutional Review Board (ID: 6365–1-9–2020). All participants provided written consent after the purpose and the procedures of the study were explained to them.

## Procedure

### Assessment

The assessment started with detailed history taking, including detailed psychiatric history and data about tinnitus side, duration, character, and course. This was followed by an otoscopic examination to ensure the external ear canal was normal and the tympanic membrane was intact. The next step was the performance of a basic audiological evaluation, extended high-frequency audiometry, psychoacoustic measures of tinnitus (tinnitus pitch and loudness matching), and assessment by the Arabic-QTR. The psychoacoustic measures and the Arabic-QTR were applied before and after the rehabilitation in both groups to evaluate the efficacy of both intervention programs.

### Basic Audiological Evaluation

Immittancemetry involved tympanometry and ipsilateral acoustic reﬂexes at 0.5, 1, 2, and 4 kHz to ensure normal middle ear function. In addition, pure-tone audiometry (PTA) was performed at frequencies ranging from 0.25 to 8 kHz for air conduction and from 0.5 to 4.0 kHz for bone conduction. Speech audiometry (speech reception threshold [SRT] and WRS) was then performed to confirm the outcomes of the PTA. The TDH-39 supra-aural headphones (Telephonics, Farmingdale, NY, United States) were used to deliver air-conduction stimuli.

### Extended High-Frequency Audiometry

The air conduction stimuli were delivered through HAD 200 circumaural headphones (Sennheiser, Wedemark, Germany) to assess the extended high-frequency measures, at frequencies of 10 and 12.5 kHz for both ears even in cases of unilateral tinnitus.

### Psychoacoustic Measures of Tinnitus

Tinnitus matching was performed in the non-tinnitus ear when tinnitus was unilateral and in the better ear when tinnitus was bilateral. 27 These measures involved pitch matching and loudness matching tests.

**Pitch matching** estimates tinnitus frequency based on the tinnitus pitch as perceived by the patient. A stimulus of 1,000 Hz was presented at 5 to 10 dB SL (regarding the SRT) followed by an octave step-up or -down movement. Half-octave steps were employed for more precise estimation after the closest frequency to the tinnitus pitch was detected. 28

**Loudness matching** is the perceived equivalent of tinnitus sound intensity. The stimulus represented frequency chosen via pitch matching that was conducted at a level just below threshold. Then, the intensity was increased in 1-dB steps until the patient signaled a match. Tinnitus loudness was measured in dBSL (regarding the SRT). 28

### The Arabic Questionnaire for Tinnitus Reaction (Arabic-QTR)

The Arabic-QTR was developed by Mekki et al. 24 to assess the severity and impact of tinnitus in adults with chronic subjective tinnitus and normal hearing. It has been proven to have adequate validity and reliability. The validity was assessed through exploratory factor analysis, with 18 items categorized into 4 subscales: 1) the somatic subscale (items 1–4), which estimates somatic complaints; 2) the awareness subscale (items 5–10), which denotes difficulties in auditory perception and physical limitations; 3) the emotional subscale (items 11–14), which represents affective and social impacts; and 4) the anxiety subscale (items 15–18), which indicates psychological symptoms. Each question has three possible answers: *yes* (2 points), *sometimes* (1 point), or *no* (0 points). A score for each subscale and a total score out of 36 can be obtained. The degree of tinnitus reaction severity can also be estimated from the total score as followd: mild – 0 to 9 (0–25%); moderate –10 to 18 (26–50%); severe –19 to 27 (51–75%); and profound –28 to 36 (> 75%).

In addition, the Arabic-QTR exhibited an adequate construct validity by revealing moderate-to-strong correlations with two validated tinnitus scales (the Tinnitus Handicap Inventory [THI] and the Mini-Tinnitus Questionnaire) 29 and a psychological assessment scale (the Arabic version of the Hospital Anxiety and Depression Scale, HADS). 30 The Arabic-QTR has also been proven to have considerable internal consistency reliability (Cronbach α coefficients [0.78], split-half reliability coefficients [0.73], and inter-item correlations) and external consistency as measured by test-retest reliability.

## Intervention

The first group received CBT, which consisted of 12 sessions over 3 months, according to the protocol suggested by Robinson et al. 31 The sessions were conducted by two expert psychiatrists who are experienced in CBT and have co-authored the current study. They involved an introduction about thoughts, behaviors, stress, and tinnitus; increasing pleasant activities; relaxation techniques; cognitive restructuring; goal setting; summary; and review. Homework assignments were given weekly and reviewed during the next session.

The second group received NST of notched white noise that has a frequency spectrum of 0.05 to 15 kHz with equal intensities. A notch is introduced within the stimulus by notched filters that are chosen to correspond to the tinnitus pitch (frequency band of 1/2 octave around the individual tinnitus frequency removed from the energy spectrum of the sound) for two hours on daily basis, through a laptop, mobile phone, or MP3 player over three consecutive months. The patients were instructed to listen to the NST attentively in a quiet surrounding at home with comfortable loudness. They were allowed reading, surfing the internet, or doing other relaxing activities. 32 In case of unilateral tinnitus, sound therapy was only applied to the symptomatic ear.

## Equipment and Materials

A two-channel audiometer (Madsen Oribter 902, version 2, Natus Medical Incorporated, Middleton, WI, United States) was used for the PTA, speech audiometry, extended high-frequency audiometry, and psychoacoustic measures of tinnitus. Moreover, a middle ear analyzer, (Madsen Zodiac 901, version 3.2, Natus Medical Incorporated) was used for immittancemetry.

## Statistical Analysis

Data obtained from all subjects were inserted into raw data tables. The statistical analysis was performed using the IBM SPSS Statistics for Windows software, version 21.0 (IBM Corp., Armonk, NY United States). Frequencies and percentages were used to express the qualitative variables, whereas mean, standard deviation (SD), range, and interquartile range values were used to express quantitative variables. The Fisher exact and Chi-squared (χ ^2^ ) tests were used to assess the distribution of the qualitative data. The Shapiro-Wilk test was applied to estimate the normality of quantitative data distribution. Parametric tests (such as Student *t* -tests) were used when the data were normally distributed, and non-parametric tests (such as the Mann-Whitney and Wilcoxon signed-rank tests) were used when data were abnormally distributed. Test significance was set at *p* < 0.05.

## Results

The two study groups showed a homogenous distribution in terms of age, gender, duration, and lateralization of tinnitus ( Table 1 ). Table 2 represents the audiological outcomes (PTA and extended high-frequency audiometry) of the ears with tinnitus, with no significant differences between the study groups. The PTA revealed bilateral normal hearing sensitivity (≤ 25 dBHL) in the frequency range from 0.25 to 8 kHz that matched the outcomes of speech audiometry. On the other hand, the extended high-frequency thresholds were abnormally elevated, with varying degrees, from mild to profound, in both ears (> 25 dB HL) in 65% of the participants at 10 and/or 12.5 kHz. All participants had bilateral type-A tympanogram with preserved acoustic reflexes, indicating normal middle ear function.

**Table 1:** Demographics and tinnitus characteristics of the two study groups

Personal criteria	CBT group ( *n* = 32)	NST group ( *n* = 32)	Test value ( *p* )
Age in years: mean ± SD (range)	34.72 ± 7.99 (21–48)	32.03 ± 8.33 (20–49)	1.316 (0.193) ^a^
Gender: male/female	13/19	14/18	0.064 (0.800) ^b^
Duration (in months)			
Mean ± SD (range)	18.58 ± 12.17 (6–48)	16.78 ± 10.32 (6–48)	0.469 (0.639) ^c^
Median (IQR)	12 (9–24)	12 (8.25–24)	
Laterality (n)			
Bilateral	19	17	2.596 (0.628) ^d^
Right ear	5	6	
Left ear	8	9	

**Abbreviations:** CBT, cognitive behavioral therapy; IQR, interquartile range; NST, notched sound therapy; SD, standard deviation.

**Notes:**^a^ Independent samples *t* -test; ^b^ Fisher exact test; ^c^ Mann-Whitney test; and ^d^ Chi-squared test.

**Table 2:** Basic audiological evaluation and extended high-frequency threshold in the two study groups (number of ears with tinnitus in the CBT group: right = 24; left = 27; in the NST group: right = 23; left = 26)

Audiological measures		CBT group (mean ± SD)	NST group (mean ± SD)	t ( *p* )
PTA thresholds (dB HL)				
0.25 kHz	Right	13.28 ± 5.02	12.03 ± 3.99	0.27 (1.10)
	Left	14.22 ± 4.23	13.75 ± 4.21	0.66(0.44)
0.5 kHz	Right	16.25 ± 3.81	15.94 ± 3.90	0.75 (0.32)
	Left	14.06 ± 4.48	13.91 ± 4.35	0.89(0.14)
1 kHz	Right	15.94 ± 4.99	15.47 ± 4.64	0.70 (0.39)
	Left	15.00 ± 3.59	15.31 ± 4.00	0.74 (0.33)
2 kHz	Right	14.69 ± 4.39	15.94 ± 4.10	0.24 (1.18)
	Left	15.47 ± 4.46	14.84 ± 4.66	0.59 (0.55)
4 kHz	Right	13.59 ± 3.42	14.06 ± 3.46	0.59 (0.55)
	Left	14.53 ± 5.29	14.53 ± 4.98	0.00 (1.00)
8 kHz	Right	16.25 ± 6.59	15.31 ± 4.74	0.52 (0.65)
	Left	16.56 ± 6.41	15.31 ± 4.91	0.38 (0.88)
SRT (dB HL)	Right	13.28 ± 3.27	12.97 ± 2.79	0.68 (0.41)
	Left	13.13 ± 3.29	13.13 ± 2.77	0.00 (1.00)
WRS (%)	Right	100	100	——–
	Left	100	100	——–
EHF thresholds (dB HL)				
10 kHz	Right	35.16 ± 19.15	35.65 ± 18.02	0.92 (0.10)
	Left	39.38 ± 20.19	40.31 ± 19.79	0.85 (0.19)
12.5 kHz	Right	42.50 ± 30.74	40.00 ± 26.04	0.73 (0.35)
	Left	47.97 ± 30.34	46.09 ± 26.99	0.80 (0.26)

**Abbreviations:** CBT, cognitive behavioral therapy; EHF, extended high frequency; NST, notched sound therapy; PTA, pure-tone audiometry; SD, standard deviation; SRT, speech reception threshold; WRS, word recognition score.

The changes in the psychoacoustic characteristics of tinnitus following CBT and NST are shown in Table 3 . In both groups, pitch matching ranged from 0.125 to 12.500 kHz bilaterally. The median values were of 3 kHz (right ear) and 4 kHz (left ear) in the CBT group, and of 1.5 kHz (right ear) and 4 kHz (left ear) in the NST group. A main finding of the present study was the stability of pitch matching outcomes throughout the therapies without any noticeable differences. However, loudness matching showed a statistically significant reduction after both therapies ( Table 3 ).

**Table 3:** Outcomes of the psychoacoustic measures pre- and posttherapy in both study groups (number of ears with tinnitus in the CBT group: right = 24; left = 27; in the NST group: right = 23; left = 26)

Psychoacoustic measures	CBT group				NST group		
	Pretherapy	Posttherapy		t ( *p* )	Pretherapy	Posttherapy	t ( *p* )
*Pitch match (Hz)*						
Right: mean ± SD (range)	3,730.20 ± 4,288.60 (125–12,500)	3,730.20 ± 4,288.60 (125–12,500)	−	3,783.20 ± 3,799.10 (125–12,500)	3,783.20 ± 3,799.10 (125–12,500)	−
Median (IQR)	3,000 (250–8,000)			3,000 (250–8,000)	1,500 (500–8,000)		1,500 (500–8,000)
Left: mean ± SD (range)	4,770.80 ± 4,769.50 (125–12,500)	4,770.80 ± 4,769.50 (125–12,500)	−	4,326.10 ± 4,114.90 (125–12,500)	4,326.10 ± 4,114.90 (125–12,500)	−
Median (IQR)	4,000 (500–4,000)			4,000 (500–4,000)	4,000 (500–9,000)		4,000 (500–9,000)
*Loudness match (dB SL)*						
Right: mean ± SD (range)	21.88 ± 3.85 (15–30)	19.17 ± 3.51 (15–25)	4.03 (0.001)*	24.13 ± 7.18 (15–35)	7.39 ± 6.55 (0–20)	15.63 (< 0.001)*
Left: mean ± SD (range)	22.59 ± 4.68 (15–35)	19.81 ± 4.69 (15–30)	5.00 (< 0.001)*	26.15 ± 8.28 (15–50)	10.00 ± 7.48 (0–30)	21.55 (< 0.001)*

**Abbreviations:** CBT, cognitive behavioral therapy; IQR, interquartile range; NST, notched sound therapy; SD, standard deviation.

**Fig. 1:**
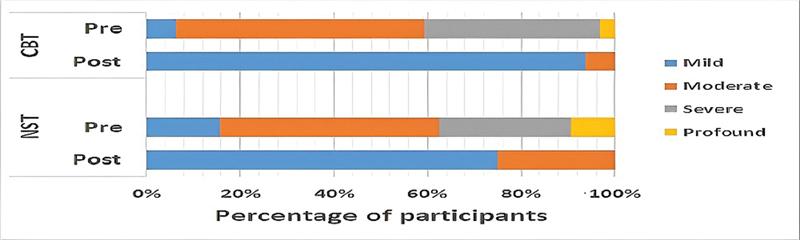
Comparison of the degree of severity in each group according to the Arabic Questionnaire for Tinnitus Reaction (Arabic-QTR). **Abbreviations:** CBT, cognitive behavioral therapy; NST, notched sound therapy.

Another important finding was the significant improvement (reduction) in the subscale and total scores on the Arabic-QTR after both therapies ( Table 4 ). Moreover, the McNemar χ ^2^ test revealed a significant reduction in the severity of tinnitus reaction in both the CBT and NST groups (CBT group: χ ^2^ *=* 4.95; *p* < 0.001; NST group: χ ^2^ = 4.79; *p* < 0.001) ( Fig. 1 ).

**Table 4:** The outcomes of the subscale and total scores on the Arabic-QTR pre- and posttherapy in both study groups

Arabic-QTR	CBT group ( *n* = 32)			NST group ( *n* = 32)		
	Pretherapy	Posttherapy	Test value ( *p* )	Pretherapy	Posttherapy	Test value ( *p* )
Somatic ^a^						
Mean ± SD (range)	4.41 ± 1.98 (0–8)	1.16 ± 0.95 (0–4)	4.79 (< 0.001)*	4.47 ± 2.36 (0–8)	1.53 ± 1.24 (0–4)	4.64 (< 0.001)*
Median (IQR)	4.5 (4–5)	1 (0.25–2)		5 (3–6)	1.5 (0.25–2)	
Awareness ^a^						
Mean ± SD (range)	6.03 ± 2.04 (1–10)	1.81 ± 1.09 (0–7)	4.97 (< 0.001)*	6.84 ± 2.99 (2–12)	2.09 ± 1.57 (0–6)	4.96 (< 0.001)*
Median (IQR)	7 (4.25–7)	2 (1–3)		6 (4–10)	1.5 (1–3)	
Emotional ^a^						
Mean ± SD (range)	3.47 ± 1.97 (0–7)	0.63 ± 1.01 (0–3)	4.95 (< 0.001)*	3.28 ± 2.28 (0–8)	1.06 ± 1.01 (0–3)	4.49 (< 0.001)*
Median (IQR)	3 (2–4.75)	0 (0–1)		3 (1–4)	1 (0–1)	
Anxiety ^a^						
Mean ± SD (range)	3.78 ± 1.66 (0–7)	0.53 ± 0.67 (0–3)	4.82 (< 0.001)*	2.88 ± 2.28 (0–7)	1.41 ± 1.29 (0–4)	3.72 (0.001)*
Median (IQR)	4 (2.25–5)	0 (0–1)		2.5 (0.25–5)	1 (0–2.75)	
Total ^b^						
Mean ± SD (range)	17.75 ± 5.72 (3–28)	4.16 ± 2.13 (1–12)	15.96 (< 0.001)*	17.34 ± 7.59 (4–32)	6.06 ± 3.62 (1–13)	14.02 (< 0.001)*

**Abbreviations:** Arabic-QTR, Arabic Questionnaire for Tinnitus Reaction; CBT, cognitive behavioral therapy; IQR, interquartile range; NST, notched sound therapy; SD, standard deviation.

**Notes:**^a^ Wilcoxon signed-rank test; ^b^ independent samples *t* -test.

**Fig. 2:**
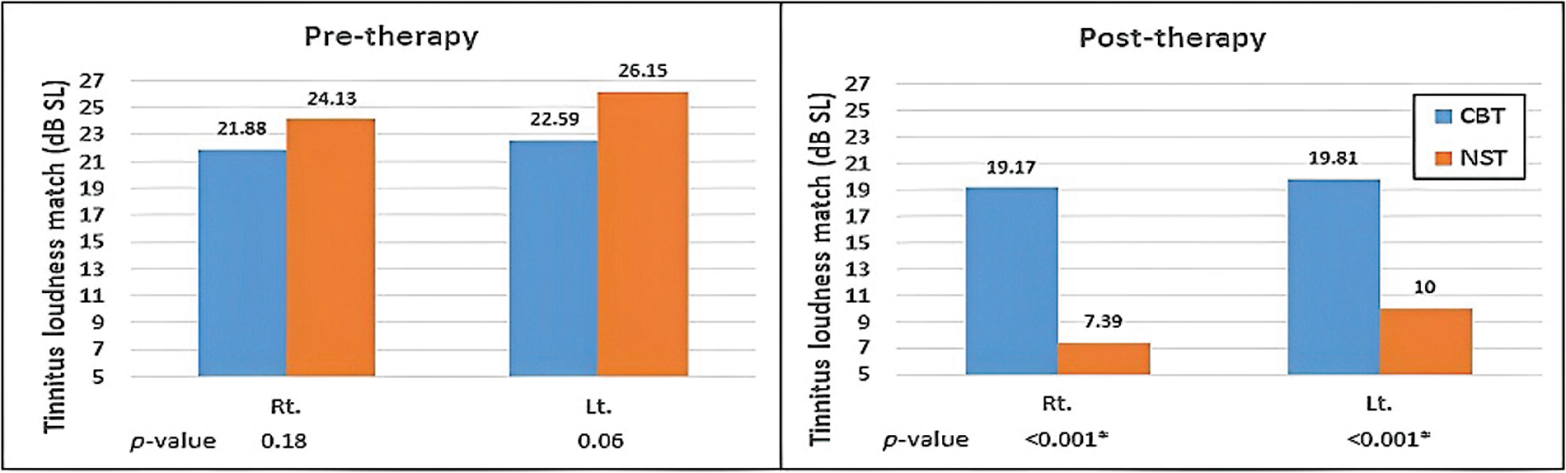
Comparison of the tinnitus loudness match between the two therapies (CBT and NST) in both ears.

**Fig. 3:**
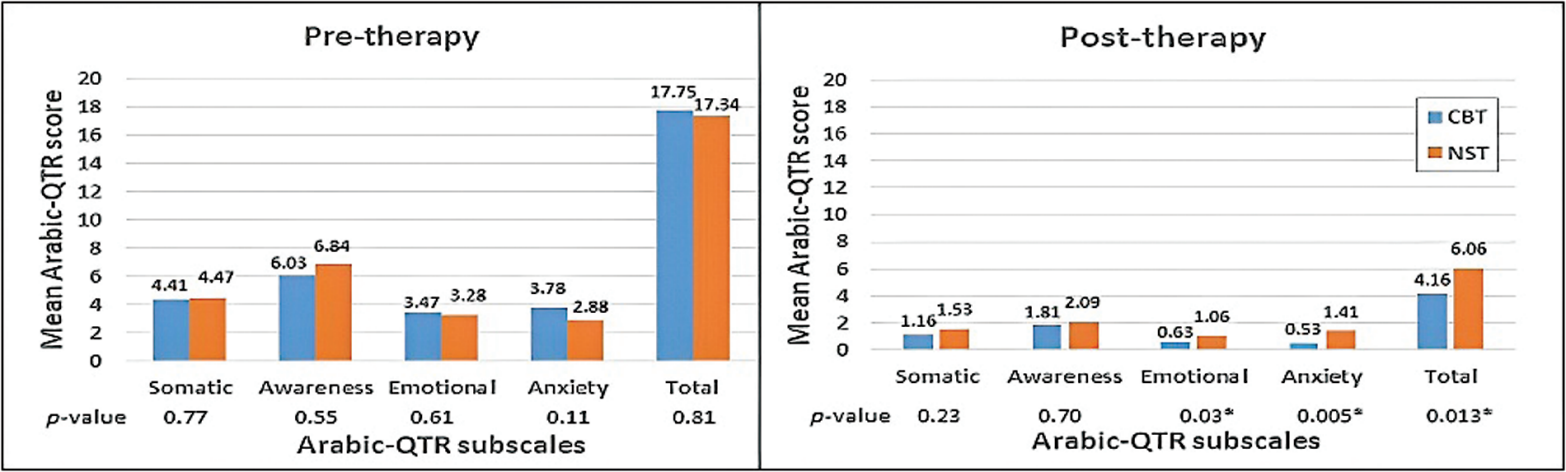
Comparison of the outcomes of CBT and NST according to the Arabic-QTR.

Moreover, the outcome measures of the two study groups were compared prior to and after the therapies. Fig. 2 shows that the loudness matching was comparable between the two study groups before therapy administration. Nevertheless, the posttherapy loudness matching revealed pronounced reduction following sound therapy as compared with the CBT using the independent samples *t* -test. When the Arabic-QTR scores were compared between the two groups, they were analogous before the intervention ( Fig. 3 ). However, there was a significant reduction in the scores on the emotional and anxiety subscales and on the total score after the CBT in comparison to the effect of noise therapy using the Mann-Whitney test ( Fig. 3 ).

## Discussion

The current study involved adults who had normal hearing sensitivity at the frequency range between 0.25 and 8 kHz, while 65% of them had varying degrees of hearing loss that ranged from mild to profound at the extended high frequencies of 10 and 12.5 kHz. Similarly, Song et al. 33 revealed that most young patients (18–35 years old) with tinnitus and normal hearing had hearing loss in the extended high-frequency region of 10, 12.5, 14, and 16 kHz. Ibraheem and Hassaan 34 found an elevated mean extended high-frequency threshold average at 10, 12.5, and 16 kHz in adults with tinnitus and normal hearing. Furthermore, Vielsmeier et al. 35 reported that 83% of adult tinnitus patients (mean age: 37.25 ± 10.25 years) with normal hearing had hearing thresholds higher than 15 dB HL at 1 or more of the 10, 11.2, 12.5, 14, and 16 kHz frequencies. Moreover, Kim et al. 36 stated that 74% of adult patients (mean age: 42.1 ± 12.8 years) with tinnitus and normal hearing at 8 kHz experienced thresholds higher than 25 dB HL at 12 kHz and/or 16 kHz.

Despite the association between tinnitus and hearing loss in 49% of individuals, 37 tinnitus may also manifest in patients with normal hearing. This could be attributed to the presence of hearing loss in the extended high frequencies or the existence of localized regions of hearing loss that cannot be examined during the routine audiometry at octave frequencies. 38 Hearing loss at the extended high-frequency region is highly age-dependent and highly susceptible to middle-ear disease, ototoxicity, noise exposure, and systemic diseases, such as cardiovascular disorders. However, it may be idiopathic without any evidence of aural pathology; 39 hence, this could provide an explanation for the extended high-frequency hearing loss in the current study, in which most of the former causes have been excluded.

One main objective of the present study was to assess CBT and NST outcomes in adults with chronic subjective tinnitus using psychoacoustic measures and the Arabic- QTR. Pitch matching remained constant after the two therapies. This could be supported by the presence of underlying pathology in this frequency region, by the findings of Neff et al,. 40 of good test-retest reliability of different methods of pitch matching, and by the fact that the notch in sound therapy is centered around a frequency based on the pitch matching. This notch is deprived of stimulation to reduce cortical activities that control it; hence, the pitch-matching frequency remains unchangeable. On the other hand, the loudness-matching test and the Arabic-QTR have been proven to be effective measures for the assessment of tinnitus and to monitor the outcomes of CBT and NST.

An important finding was the significant improvement in loudness perception (reduction in the loudness-matching level) and tinnitus reaction (lower scores on the Arabic- QTR,) as perceived by patients in both groups. In the CBT group, the reduction of the level of tinnitus loudness after CBT agreed with the results of Andersson and Lyttkens, 41 who examined the effect of CBT on tinnitus and found that tinnitus loudness became weaker or even disappeared with a considerable reduction in tinnitus annoyance at the follow-up assessment.

In addition, the current study revealed an improvement in tinnitus reaction as measured by the Arabic-QTR subscale and total scores after CBT. The participants exhibited mild, moderate, severe, and profound degrees of tinnitus reaction, as measured by the Arabic-QTR before CBT, whereas, after CBT, the degrees became better: mild in more than 80% and moderate degrees only. Similarly, Robinson et al. 31 revealed a significant reduction in the global tinnitus severity as measured by a QoL questionnaire specific for tinnitus and the Tinnitus Handicap Questionnaire. Also, Martinez Devesa et al. 9 investigated the role of CBT in tinnitus management and found that QoL scores and depression scores were improved in tinnitus patients; contrary to the results of the current study, they reported no effect of CBT on tinnitus loudness. Consequently, CBT has been considered an effective treatment for tinnitus-related distress, in addition to its moderate-to-strong effect on tinnitus-related QoL.

Of particular importance, the effect of NST caused a remarkable decrease in tinnitus loudness level and a notable improvement in tinnitus distress, as reflected by the reduction in the Arabic-QTR subscale and total scores posttreatment and the increase in tinnitus reaction severity from mild, moderate, severe, or profound before NST to mild (in more than 75% of the participants) and moderate degrees only after the therapy. These results agree with those of Wunderlich et al., 42 who demonstrated that three months of listening to customized sound therapy had led to a decrease in tinnitus loudness. Additionally, Therdphaothai et al., 10 Zhang, 11 Mahboubi et al., 12 and Okamoto et al. 13 reported a favorable effect of NST on the perceived annoyance and tinnitus handicap. In line with the results of the present study, He et al. 43 found that customized sound therapy (music) had efficiently reduced the THI scores of tinnitus patients and ameliorated the negative effects and distress caused by chronic tinnitus. Also, Wang et al. 44 evaluated the effect of customized sound therapy (music) and found a significant improvement in THI and HADS scores. The effect of sound therapy in these studies and in the present study is based on the adaptation level theory, in which sound therapy facilitates the process of habituation of both tinnitus-induced reaction and tinnitus perception by decreasing the difference between tinnitus-related neuronal activity and background neuronal activity. 45

Another fundamental objective of the present study was to compare the outcomes of assessment measures between the two therapies. The NST was found to yield more improvement in tinnitus loudness perception when compared with the CBT. Conversely, tinnitus distress was more reduced after CBT, as reflected by the lower scores on the emotional and anxiety subscales and the total score on the Arabic-QTR in comparison to the NST group. These results are not surprising, as NST focuses more on decreasing the loudness of tinnitus, whereas CBT acts mainly on reducing tinnitus-related distress. There is a vicious circle involving tinnitus loudness and tinnitus-related annoyance and distress. 6 Consequently, when tinnitus loudness is lowered (the target of NST), tinnitus-related distress will be reduced. On the other hand, when tinnitus distress is reduced (the target of CBT), this will surely be reflected in a decrease in tinnitus loudness. Therefore, a combination of CBT and NST can be recommended as a tinnitus management program in chronic subjective tinnitus to provide favorable outcomes both physically and psychologically.

## Limitations

Although the sample size was calculated to determine the number of participants, a larger sample could have provided more reliable data. Moreover, some patients may become reluctant to continue the CBT because of its multiple sessions, in addition to the difficulty in adjusting and following up the home-based NST in some cases. However, a unique aspect of strength is that the present work constitutes the first to compare the effects of CBT and NST in the management of adults with chronic subjective tinnitus.

## Conclusion

The current study revealed the effectiveness of CBT and NST in the management of tinnitus-related distress and tinnitus loudness as assessed by the Arabic-QTR and tinnitus loudness matching in pre and posttherapy sessions. In a comparison of the effect of the two therapies, CBT was found to be more effective in decreasing tinnitus-related distress than NST, whereas NST was found to be more helpful in decreasing tinnitus loudness than CBT. Combining these two treatment approaches should be considered to obtain optimal outcomes.
